# Towards a more molecular taxonomy of disease

**DOI:** 10.1186/s13326-017-0134-0

**Published:** 2017-07-27

**Authors:** Jisoo Park, Benjamin J. Hescott, Donna K. Slonim

**Affiliations:** 1 Department of Computer Science, Tufts University, 161 College Avenue, Medford, 02155 MA USA; 2 Department of Integrative Physiology and Pathobiology, Tufts University School of Medicine, 145 Harrison Avenue, Boston, 02111 MA USA

**Keywords:** Disease Ontology inference, Disease tree inference, Pairwise disease similarity, Disease gene association, Medical Subject Headings tree, Disease Ontology, Hierarchical clustering, Parent Promotion

## Abstract

**Background:**

Disease taxonomies have been designed for many applications, but they tend not to fully incorporate the growing amount of molecular-level knowledge of disease processes, inhibiting research efforts. Understanding the degree to which we can infer disease relationships from molecular data alone may yield insights into how to ultimately construct more modern taxonomies that integrate both physiological and molecular information.

**Results:**

We introduce a new technique we call Parent Promotion to infer hierarchical relationships between disease terms using disease-gene data. We compare this technique with both an established ontology inference method (CliXO) and a minimum weight spanning tree approach. Because there is no gold standard molecular disease taxonomy available, we compare our inferred hierarchies to both the Medical Subject Headings (MeSH) category C forest of diseases and to subnetworks of the Disease Ontology (DO). This comparison provides insights about the inference algorithms, choices of evaluation metrics, and the existing molecular content of various subnetworks of MeSH and the DO. Our results suggest that the Parent Promotion method performs well in most cases. Performance across MeSH trees is also correlated between inference methods. Specifically, inferred relationships are more consistent with those in smaller MeSH disease trees than larger ones, but there are some notable exceptions that may correlate with higher molecular content in MeSH.

**Conclusions:**

Our experiments provide insights about learning relationships between diseases from disease genes alone. Future work should explore the prospect of disease term discovery from molecular data and how best to integrate molecular data with anatomical and clinical knowledge. This study nonetheless suggests that disease gene information has the potential to form an important part of the foundation for future representations of the disease landscape.

**Electronic supplementary material:**

The online version of this article (doi:10.1186/s13326-017-0134-0) contains supplementary material, which is available to authorized users.

## Background

The recent growth in availability of genomic and clinical data allows for the discovery of new molecular-level mechanistic models of disease. However, existing disease taxonomies and ontologies are often focused on either physiological characterizations of disease, sometimes using decades-old criteria, or on the organizational and billing needs of hospitals. Automatically inferring common molecular links between related diseases is made more difficult by the limited molecular representation in current taxonomies [1], leading some researchers to manually group related disorders for individual projects (for example, PheWAS analysis [2] or network-based disease gene prioritization [3]). Yet such manual efforts limit consistency and reproducibility. To further advance such research and biomedical knowledge in the genomic era, a recent National Academy of Sciences working group has called for the development of new disease taxonomies better suited to incorporate molecular information [4].

A truly modern taxonomy would presumably combine clinical, physiological, and molecular data. The question we address here is the degree to which we can infer a meaningful disease taxonomy simply using disease gene information. In this, we were inspired by efforts by Trey Ideker’s group to infer a version of the Gene Ontology using pairwise similarity scores between genes [5, 6]. Their CliXO algorithm, for example, sorts gene pairs by a pairwise similarity score and incrementally uses these scores to group together cliques of similar genes. The resulting ontology forms a Directed Acyclic Graph (DAG) of sets of genes. As in that work, here we are *not* arguing that we should ultimately construct a disease hierarchy automatically in this way. However, learning how we can discover the relationships in existing disease taxonomies from disease gene data is a first step towards developing new hierarchies of disease that integrate the clinical information used in today’s taxonomies with genomic data. Such integrated taxonomies are needed to better support research in molecular medicine [7].

To infer a disease taxonomy, we would like to simply cluster diseases hierarchically based on associated genes from a large gene-disease database. However, if the items we are clustering are diseases, the internal nodes of any hierarchical clustering method will correspond to unnamed sets of diseases. While some of these may be informative, identifying them is a challenge. We therefore introduce here an algorithm called Parent Promotion, based on hierarchical clustering, that addresses this problem.

We acknowledge that we are deliberately blurring the distinction here between an ontology of disease [8] and a disease taxonomy [9]. In this manuscript, we focus on learning a hierarchical characterization of disease using existing disease terminology, yet incorporating molecular relationships. Such a description may be able to better identify novel relationships between disorders that do not appear clinically similar but that arise from similar underlying genotypes. Yet we are not expecting here to comprehensively infer disease relationships as in most ontologies, in part because the current project ignores the clinical and anatomical characteristics built into many existing taxonomies. Accordingly, we frequently use the term “disease hierarchy” to encompass our inferred hierarchies as well as those to which we compare.

One important question is how to evaluate our inferred hierarchies of disease when there is no existing gold standard. However, there are a handful of existing taxonomies and disease ontologies that are somewhat suitable for molecular analyses and comparisons [4]. Medical Subject Headings (MeSH) is a hierarchical structure of controlled biological vocabularies used to index articles in MEDLINE [10]. MeSH includes many medical concepts beyond diseases, but here we refer to MeSH category C, a comprehensive set of 26 trees that represent relationships between diseases. SNOMED-CT provides an organized terminology for clinical terms [11]; this is one of the most detailed terminologies available, but there are restrictions on its distribution. The Unified Medical Language System (UMLS) metathesaurus includes disease terms from multiple taxonomies; while it is not intended to be an ontology, its semantic network can identify some relationships between terms [12]. The Disease Ontology (DO) also integrates the knowledge and relationships from several taxonomies, including MeSH, SNOMED-CT, and ICD [13].

Initially, because of the high coverage and availability of MeSH and its simple structure, we chose to compare our inferred hierarchies to the MeSH forest of disease terms. Although it is not necessarily a gold standard for the problem we are trying to solve, we can use such a comparison to identify the strengths and limitations of different inference methods. In addition, identifying individual MeSH disease trees that are more consistent with the hierarchies inferred from disease-gene data helps in assessing the molecular content of existing domains in MeSH. We have also extended our assessments by comparison to the Disease Ontology, which is a more complex process for reasons detailed below.

Even after fixing a “reference” hierarchy for comparison, the question of how to assess correctness remains. Many of the standard network and graph comparison metrics are inappropriate for our problem. One that does make sense is a strict variant of Edge Correctness [14] that asks how many parent-child relationships we get right. We therefore use Edge Correctness as one measure of accuracy.

One limitation of Edge Correctness, however, is that the distances between pairs of terms are not uniform [15]. That is, two diseases that are separated by more than one taxonomic link may be more closely related to each other than two other diseases in a direct parent-child relationship. We therefore also introduce the notion of Ancestor Correctness, a feature-based similarity measurement [16] that assesses our ability to properly identify ancestry without concern about distances.

Finally, neither Edge Correctness nor Ancestor Correctness penalizes an algorithm for false positives (inferred edges not in the reference hierarchy). This is fine for inference methods like Parent Promotion that build trees, which all have the same number of edges for a fixed set of disease nodes, but not for comparison to ontology-learning approaches that can add arbitrary numbers of edges. Accordingly, we also compute a variation of hierarchical precision and recall [17], analagous to Ancestor Correctness, that accounts for both false positives and false negatives.

## Methods

### Reference taxonomies

To quantify performance of various disease hierarchy inference methods, we compare our inferred taxonomies to the 2016 Medical Subject Headings (MeSH) disease trees [10] and the Disease Ontology (DO) [18], downloaded on August 5, 2016. From both datasets, we exclude diseases for which we cannot find any associated genes, because our methods would then have no way to learn about how they relate to other diseases. However, excluding diseases can disconnect our reference hierarchies. To reconnect them, we therefore add edges from a deleted node’s parents to all of its closest descendants that do have associated genes.

We note that the MeSH trees allow repeated disease names, resulting in multiple nodes with the same name in different parts of the tree. We treat these terms as if they were the same node, effectively matching against the corresponding DAG. However, given that the original structure is a tree, most of these DAGs end up being fairly tree-like.

Because the Disease Ontology is substantially larger than any of the individual MeSH trees, we extracted smaller DAGs from the full DO to facilitate algorithm comparison. To find these smaller DAGs, we searched through the DO starting at the most general term. A term became a root of a DO subnetwork if its name approximately corresponded to the name of the root of one of the 26 MeSH trees and if it had at least 100 DO terms as descendants. This approach identified four new DAGs that can be described as covering mostly “Cardiovasular Disease,” ”Gastrointestinal Disease,” “Musculoskeletal Disease,” and “Nervous System Disease”.

Table 1 reports the sizes and topology of these four subnetworks of the DO. All are fairly tree-like; only small numbers of nodes have more than one parent, and the total number of edges is not that much larger than the number of nodes. We note that it is not necessarily the case that all disease nodes in the DAG labeled Musculoskeletal Disease, for example, actually correspond to musculoskeletal disorders, because the Disease Ontology and MeSH are organized according to different principles. We therefore acknowledge that each subnetwork of the DO may contain terms that map to several different MeSH disease trees. Nonetheless, we use these labels as shorthand ways to refer to the chosen DO subnetworks.

**Table 1:** Subnetworks of the Disease Ontology

Root disease	#Diseases (nodes)	#Edges	#Nodes with 1 parent	#Nodes with 2 parents	#Nodes with 3 parents
Disease	2,039	2,095	1,982	55	1
Cardiovascular disease	141	141	139	1	0
Gastrointestinal disease	115	118	110	4	0
Musculoskeletal disease	133	135	129	3	0
Nervous System disease	308	324	291	15	1

The entire Disease Ontology (root = “Disease”) and four subnetworks of various sizes extracted from it. The original DO and its subnetworks are tree-like: 1) the numbers of edges are close to *n*−1, where *n* is the number of nodes and 2) only a small fraction of nodes have 2 or more parents

### Withheld MeSH subtrees for method development

We selected four small subtrees from MeSH that we used for refining our computational methods. These are the MeSH subtrees rooted at the terms “Infant Premature Diseases,” “Dementia,” “Respiration Disorders,” and “Eye Diseases,” giving us a range of subtrees of different sizes and complexity (Table 2). Note that the MeSH tree rooted at “Eye Diseases” includes 149 disease terms and 178 edges, indicating that several terms appear multiple times, although we allow a node with a given name to appear only once in each inferred hierarchy.

**Table 2:** Four MeSH subtrees of various sizes used for method development

Root disease	#Diseases (nodes)	#Edges
Infant, Premature, Diseases	6	5
Dementia	13	12
Respiration disorders	23	22
Eye diseases	149	178

Although we show the performance of the inference methods on these subtrees separately in Additional file 1, we did not think it fair to include them in our overall MeSH results because we used them to tune our methods. Accordingly, we removed the subtrees rooted at these nodes from the relevant disease trees in MeSH before evaluating the different methods’ performance. Only one whole disease tree, C11 (“Eye Diseases”), was removed, because the entire C11 tree was used for method development.

There are two other MeSH disease trees that were also removed before evaluation: C21, “Diseases of Environmental Origin,” which included only 3 diseases with associated genes, and C22, “Animal Diseases,” which contained no diseases with associated genes. We therefore report averaged MeSH results over the remaining 23 MeSH disease categories.

### Disease genes

We use disease genes to calculate pairwise similarity of diseases. For our comparison to MeSH, we gathered disease-gene associations from the Online Mendelian Inheritance in Man (OMIM) database [19] and the Genopedia compendium in the HuGE database of Human Genetic Epidemiology [20], both downloaded on February 3rd, 2016. OMIM contains human genes, phenotypes (typically specific diseases), and information about relationships between them. In particular, OMIM phenotypes include Mendelian disorders, whose associated genes are either known or not yet known, as well as mutations that increase susceptibility to infection, cancer, or drugs [21]. Genopedia includes links to articles on epidemiological studies that identify gene-disease interactions. The majority of these are discovered through association studies; linkage mapping and animal studies are specifically excluded [20]. We combined disease-gene associations from the two databases as in our previous work [1], using the MEDIC merged disease vocabulary (downloaded from the Comparative Toxicogenomics Database [22] on February 3rd, 2016). This combined data set contains 2755 diseases and 12,873 genes.

To infer hierarchies based on DO terms with this disease-gene data, however, required converting the MeSH disease terms to DO terms. The DO obo file provides synonym information for this conversion. However, because not every MeSH term has a DO equivalent, nor vice-versa, the mapped disease gene data set included 1790 DO terms with 12,230 associated genes. The Disease Ontology actually includes 6932 disease nodes, so the resulting DAG of diseases with associated genes was largely disconnected.

For the DO analysis, we therefore augmented the disease gene data with disease-gene associations from the DISEASES database [23] (downloaded on August 5th, 2016) which directly uses DO terms. We used the filtered version of the DISEASES database which provides non-redundant disease-gene association pairs, and selected only associations derived from experiments or database curation (“knowledge”), which we expect to be of relatively high confidence. The DISEASES data included 772 disease terms and 13,059 genes. When combined with the mapped data from the MeSH comparison, the total yielded 2039 DO terms with 16,404 associated genes, producing a sufficiently connected ontology for our purposes.

Although this number of disease genes seems high, note that our “genes” are really referring to entities with distinct HGNC “official gene symbols,” as reported in the NCBI Gene database and associated with some disease term in the databases described. Some HGNC symbols refer to distinct subunits of genes, while a few (under 3.5%) refer to non-coding sequences that have either been shown to play a regulatory role in disease, or that are locations of SNPs linked to disease in GWAS studies. At most 250 such non-coding entities are implicated in more than one disease and might therefore potentially play a role in our analyses.

### Measuring pairwise similarity

For our inference algorithms we need methods to measure similarities both between pairs of diseases and between pairs of genes. To calculate pairwise similarity between diseases *A* and *B*, *disease*_*sim*(*A,B*), let *G*_*A*_ be the set of associated genes for disease *A* and *G*_*B*_ the set of associated genes for disease *B*. We then use the Jaccard Index [24] to represent the similarity between the disease gene sets as follows:

$$ {disease\_sim(A,B)=Jaccard(G_{A},G_{B})={\arrowvert{G_{A}\cap G_{B}}\arrowvert \over \arrowvert{G_{A}\cup{G}_{B}}\arrowvert}} $$

To calculate pairwise similarity between genes *g*_1_ and *g*_2_, *gene*_*sim*(*g*_1_,*g*_2_), we do the opposite, as we are interested in measuring the similarity of diseases with respect to their associated genes:

$$ {gene\_sim(g_{1},g_{2})=Jaccard(D_{g_{1}},D_{g_{2}})={\arrowvert{D_{g_{1}}\cap{D}_{g_{2}}}\arrowvert \over \arrowvert{D_{g_{1}}\cup{D}_{g_{2}}}\arrowvert}} $$

where $D_{g_{1}}$ is the set of diseases associated with gene *g*_1_ and $D_{g_{2}}$ is the set of diseases associated with gene *g*_2_.

Note that no information about the relationships between diseases other than this measure of overlapping disease genes is incorporated into this similarity matrix or used by our inference algorithms.

### Inference strategies

#### Clique Extracted Ontology (CliXO)

To use CliXO to generate disease ontologies, we begin by creating a matrix containing the Jaccard similarity score between genes as defined above. CliXO uses this similarity matrix as input. It also relies on two parameters: *α*, which represents the amount of noise allowed in forming cliques, and *β*, which represents missing data. The algorithm is demonstrated to be relatively robust to variation in *β*, so we set *β*=0.5 as done by the CliXO team [5]. Variation in *α* has higher impact on the results, so tuning it to the data set is suggested. We chose *α*=0.05 because it produced reasonable-sized output graphs in our initial experiments on the four MeSH subtrees in Table 2.

**Fig. 1:**
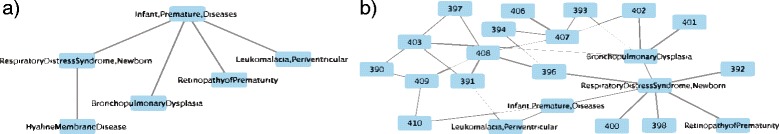
Topological difference between MeSH and the corresponding inferred ontology using CliXO. **a** A MeSH subtree containing prematurity complications. **b** Corresponding Disease Ontology inferred using CliXO and ontology alignment. Drawn in Cytoscape v. 3.3.0 [30]

Initially, CliXO returns a DAG whose internal nodes correspond to sets of genes, not to specific disease terms in the reference ontology. We then used the ontology alignment technique of [6] to align the resulting ontology to the MeSH reference or to the Disease Ontology, in order to identify disease terms in the output DAG. Accordingly, some of the disease terms may not be represented in the CliXO output, because they fail to map to any node. (Fig. 1 demonstrates the topological difference for a small example; note that the CliXO output on the right maps only 5 of the 6 disease nodes.)

#### Parent Promotion

We introduce a new technique we call Parent Promotion that focuses on similarities in disease genes. The idea is to group diseases by their similarity scores and use hierarchical clustering to form subgroups. Parent-child relations are then created from these subgroups by counting citation frequency in PubMed.

Specifically, we transform the pairwise similarity score into a distance by subtracting it from 1. We then perform complete-linkage hierarchical clustering on the disease terms using the hclust function in R with these distances. Internal nodes in this dendrogram correspond to sets of diseases. To convert the resulting dendrogram to a hierarchy with a single disease at each node, we identify the number of disease-related articles in PubMed for each disease in a cluster using the NCBI’s E-utilities (http://www.ncbi.nlm.nih.gov/books/NBK25501/).

**Fig. 2:**
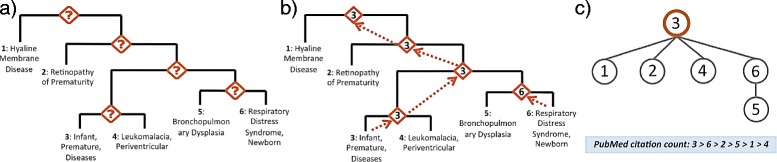
How the Parent Promotion method transforms a dendrogram created by hierarchical clustering. **a** Dendrogram for diseases of infants born preterm. Hierarchical clustering builds a tree whose internal nodes are hard to interpret. **b** Parent Promotion finds the most general disease term from each cluster and promotes it as an internal node. An internal node becomes the parent of all other nodes in the same cluster. Disease term 3 has the most citations and keeps being selected for promotion until it becomes the root. Disease term 6 has more citations than 5 and is promoted as the parent of 5. However, it later becomes a child of 3 because it has fewer citations than 3. **c** Final tree built by Parent Promotion

Working up from the bottom of the dendrogram, the disease term with the most citations is promoted to become the parent, with all other diseases in the cluster left as its children. Once defined as a child, a disease does not have another chance to be promoted. That is, we only consider the most recently promoted disease and its siblings in a cluster when deciding the next parent. Figure 2 shows an example of how the dendrogram guides the Parent Promotion process.

Notice that the inferred tree created by the Parent Promotion technique always has the same number of diseases (nodes) as the reference. However, the number of edges may differ from that of the reference, which may be either implicitly or explicitly a DAG. In either case, Parent Promotion may therefore produce a result with fewer edges.

#### Minimum weight spanning tree

We also compared our new Parent Promotion method to the standard technique of finding a Minimum Weight Spanning Tree (MWST) [25] over the complete network of disease terms, with pairwise similarity scores between diseases as edge weights. The idea behind this is that a representation of the relationships between diseases that connects all the disease terms by their highest disease gene similarity represents a minimum-length description of the data that seems likely to capture real disease relationships. The MWST is unrooted, so we choose the disease with the most related PubMED articles as the root.

### Evalution metrics

Comparing the inference methods remains challenging due to the topological differences of the output. In particular, both Parent Promotion and MWST produce trees whose *n* nodes are exactly those of the reference hierarchy. In contrast, the DAG output by the CliXO method may be much larger (as in Fig. 1). We use multiple methods to quantify and compare performance despite these differences.

#### Edge Correctness (EC)

Inspired by the notion of Edge Correctness (EC) used in network alignment [14] we measure the number of edges that are identical to those in the reference hierarchy. Unlike in the network alignment problem, which uses Edge Correctness as a proxy for node correctness, for this problem we know the node correctness and wish to measure correctly inferred edges. We count edges as correctly matched if and only if the parent child relations (both the edges and the directions of the edges) are preserved. To create an overall score we calculate the percentage of edges in the reference that also appear in the inferred ontology.

#### Ancestor Correctness (AC)

While Edge Correctness (EC) can measure how well two networks are aligned, it may not be the best method for evaluating disease taxonomies. In particular, diseases separated by multiple taxonomic links may still be closely related to each other, so EC can underestimate performance by ignoring the ancestor-descendant relationship. EC also rewards successfully matched edges with no penalty for incorrect ones. This property may favor CliXO, which tends to produce DAGs with many edges.

To address the first shortcoming, we introduce the notion of Ancestor Correctness (AC). For a disease *x*, let *x*_*ref*_ be a node representing *x* in the reference ontology and *x*_*inf*_ be a node representing *x* in our inferred hierarchy. Also let *A*(*x*) be the set of all ancestors of *x* in the appropriate hierarchy. Then for a specific disease *x*_*inf*_ in the inferred taxonomy we can measure how well it matches the reference by calculating *Ancestor Jaccard = Jaccard*(*A*(*x*_*ref*_),*A*(*x*_*inf*_)). We can then apply *Ancestor Jaccard* globally by averaging across all diseases in the inferred network. We report this average as our AC score for the inferred network. Note that we only consider diseases existing in both hierarchies. However, we exclude diseases that are roots in both because they do not have any ancestors.

#### Ancestor Precision and Recall (AP and AR)

Ancestor Correctness (AC) provides a good estimate of topological similarity in terms of the number of preserved ancestors of mapped nodes. However, it still does not penalize false positives.

To address this problem, we adapt the Hierarchical Precision (*HP*) and Hierarchical Recall (*HR*) measurements from Verspoor et al. [17]. These measurements compare the sets of all ancestors of a disease in the inferred hierarchy to the ancestors of the same term in the reference. Informally, *HP* is the fraction of *x*’s ancestors in the inferred hierarchy that are correct, while *HR* is the fraction of true ancestors of *x* that are also predicted by an inference method to be ancestors of *x*.

More specifically, for a disease *x*, let *x*_*ref*_ be the node in the reference and *x*_*inf*_ be the node in the inferred ontology. Then our *HP* and *HR* are calculated as follows:

1 $$ HP(x_{ref}, x_{inf}) = {\arrowvert{A(x_{ref}) \cap A(x_{inf})}\arrowvert \over \arrowvert{A(x_{inf})}\arrowvert}  $$

2 $$ HR(x_{ref}, x_{inf}) = {\arrowvert{A(x_{ref}) \cap A(x_{inf})}\arrowvert \over \arrowvert {A(x_{ref})}\arrowvert}  $$

We also calculate an *F* score using *HP* and *HR* as:

3 $$ F(x) = 2 \times {HP(x) \times HR(x) \over HP(x) + HR(x) }  $$

Finally, we define Ancestor Precision (*AP*) and Ancestor Recall (*AR*) to be the average of *HP* and *HR* across all diseases in our reference hierarchy.

## Results

### Comparison to MeSH

We ran all three algorithms on the disease gene data and disease terms from each of the 23 MeSH trees. Table 3 reports the averaged performance across all 23 trees for each method and the different evaluation criteria. Across this data set, we see that Parent Promotion on average outperforms CliXO and MWST for almost all evaluation measures. The only exception is Ancestor Recall, for which MWST slightly edges out Parent Promotion. Detailed performance on each MeSH disease tree is shown in Additional file 1; in most cases the methods’ relative performance is similar to that in Table 3. The detailed table also shows that, for each evaluation criterion, performance of the different methods is highly correlated across the 23 disease trees, suggesting that some trees are more consistent with the disease gene data than others.

**Table 3:** Average performance of inference methods across the MeSH trees

Method	EC (± stdev)	AC (± stdev)	AP (± stdev)	AR (± stdev)	F (± stdev)
Parent Promotion	*0.13* (±*0.06*)	*0.30* (±*0.10*)	*0.46* (±*0.16*)	0.47 (± 0.14)	*0.47* (±*0.15*)
CliXO	0.12 (± 0.10)	0.22 (± 0.12)	0.30 (± 0.14)	0.38 (± 0.17)	0.33 (± 0.15)
MWST	0.07 (± 0.04)	0.11 (± 0.07)	0.13 (± 0.08)	*0.48* (±*0.18*)	0.21 (± 0.11)

Average Edge Correctness (EC), Ancestor Correctness (AC), Ancestor Precision (AP), Ancestor Recall (AR) and F-score across the different trees in the MeSH forest. Standard deviation is shown in parentheses. Best performance across different inference techniques is highlighted in italic

### Comparison to the Disease Ontology

We first attempted to reconstruct all of the Disease Ontology reflected in our disease-gene data set (2095 edges connecting 2039 DO terms). However, we could not compare the performance of all three inference methods on this full data set because running CliXO, which has at its core the computationally hard problem of finding cliques, was infeasible on a data set this large and complex. Nonetheless, we found that Parent Promotion consistently outperformed MWST on this large data set. Specifically, Parent Promotion had an EC of 0.07 compared to MWST’s EC of 0.05, an AC of 0.23 compared to MWST’s AC of 0.04, and an F score of 0.40 compared to MWST’s 0.08.

We used the subnetworks of DO listed in Table 1 to compare all three methods. Table 4 shows the results of all three methods on these subnetworks of DO. We again see that in most cases Parent Promotion outperforms CliXO and MWST for each evaluation measure, with the exception of “Musculosketal Disease,” where CliXO outperforms Parent Promotion and MWST. Again, MWST often has good Ancestor Recall despite unimpressive performance on most other metrics.

**Table 4:** Evaluation results for four DO subnetworks

	Edge Correctness			Ancestor Correctness			F-score (Ancestor precision, ancestor recall)		
	Parent			Parent			Parent		
Root disease	Promotion	CliXO	MWST	Promotion	CliXO	MWST	Promotion	CliXO	MWST
Cardiovascular disease	0.06	*0.09*	0.07	*0.32*	0.18	0.11	*0.50*	0.27	0.21
							(*0.57*, 0.44)	(0.24, 0.30)	(0.13, *0.48*)
Gastrointestinal disease	*0.17*	0.13	0.03	*0.37*	0.26	0.14	*0.55*	0.39	0.26
							(*0.56*, *0.53*)	(0.36, 0.42)	(0.18, 0.48)
Musculoskeletal disease	*0.16*	0.08	0.10	0.15	*0.26*	0.09	0.26	*0.41*	0.17
							(*0.44*, 0.18)	(0.42, *0.40*)	(0.16, 0.19)
Nervous System disease	*0.13*	0.07	0.09	*0.29*	0.17	0.10	*0.46*	0.30	0.19
							(*0.70*, 0.34)	(0.26, 0.34)	(0.13, 0.34)

Average Edge Correctness (EC), Ancestor Correctness (AC), Ancestor Precision (AP), Ancestor Recall (AR) and F-score across four DO subnetworks. Standard deviation is shown in parentheses. Best performance across different inference techniques is highlighted as italic

**Fig. 3:**
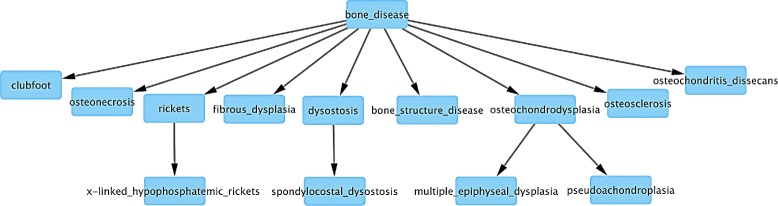
Parent Promotion tree using DO data. Subtree of the disease tree built by Parent Promotion on DO “musculoskeletal system disease” data that is an exact match to nodes and edges in the DO

Figure 3 shows an example of one of the larger connected components inferred by Parent Promotion using the DO data. All edges in the figure occur in both the Disease Ontology and the inferred tree. Although the inferred tree is relatively flat, the figure demonstrates that inference method is capturing some logical relationships between diseases.

### Data sources and quantity matter

We investigated the influence of the type and amount of data using Parent Promotion on the MeSH disease trees. First, we tried using data from just OMIM or just Genopedia. OMIM has a higher percentage of monogenic diseases identified using classical methods such as positional cloning, while Genopedia has a higher percentage of GWAS data. On the other hand, OMIM includes much *less* data, containing just 2434 genes linked to 1173 disorders, whereas Genopedia contains 12,527 genes implicated in 2499 disorders. Therefore, it is not surprising that performance on the Genopedia data exceeds that on the OMIM data, nearly across the board. The exception, interestingly, is C16, “Congenital, Hereditary, and Neonatal Diseases and Abnormalities,” where the OMIM-only version outperforms Genopedia-only by the AC, AP, and F measures. This seems likely to be because this MeSH tree includes many hereditary disorders whose genes are particularly likely to be included in OMIM. Detailed results for this comparison appear in Additional file 2. (EC is omitted because it is uninformative for many of the smaller data sets.)

In most cases, furthermore, the combination of the two data sources is better than either alone. There are a few cases where performance declines slightly with both compared to just Genopedia, but in those cases the OMIM data actually adds just a handful of genes that aren’t already in the Genopedia data, and the changes in performance are small, consistent with small random perturbations.

To further explore the hypothesis that more data produces better results, we also ran an experiment where we randomly removed 25% or 50% of the disease-gene associations from each MeSH tree, and again tried to infer trees via Parent Promotion. On average, performance on all measures improved with more data, although the effects on most individual trees were modest (results are in Additional file 3).

## Discussion

Overall, these experiments have provided some important insights into what can and cannot be learned about disease relationships from disease genes alone.

The correlations observed across the MeSH trees suggest that disease relationships in some MeSH categories are easier to learn than others. Correctness appears to be higher for smaller trees, perhaps simply because there are fewer possibilities. However, there are some large disease subtrees with higher AC and EC scores, especially Endocrine System Diseases (C19), Nutritional and Metabolic Diseases (C18), and Respiratory Tract Diseases (C08).

It is possible that the MeSH hierarchy in these areas is better defined by molecular data, or that there are simply more disease genes known in these areas than in some others. One observation is that these categories include several well-studied complex diseases with high public health impact. For example, C19 includes diabetes and ovarian and pancreatic cancer; C18 also includes diabetes, plus obesity and related conditions; and C08 features asthma, COPD, and several types of lung cancer. Which exact properties of a set of diseases contribute most to the success of inference algorithms is an important question for future work.

On the “Musculoskeletal Disease” DO subnetwork, CliXO outperforms Parent Promotion by several criteria. Parent Promotion struggles with this region of the Disease Ontology, in part because the term “Musculosketal Disease” has fewer PubMed citations than the less general term “Bone Disease.” The latter is therefore promoted incorrectly to become the root, while the former remains low in the inferred tree.

**Fig. 4:**
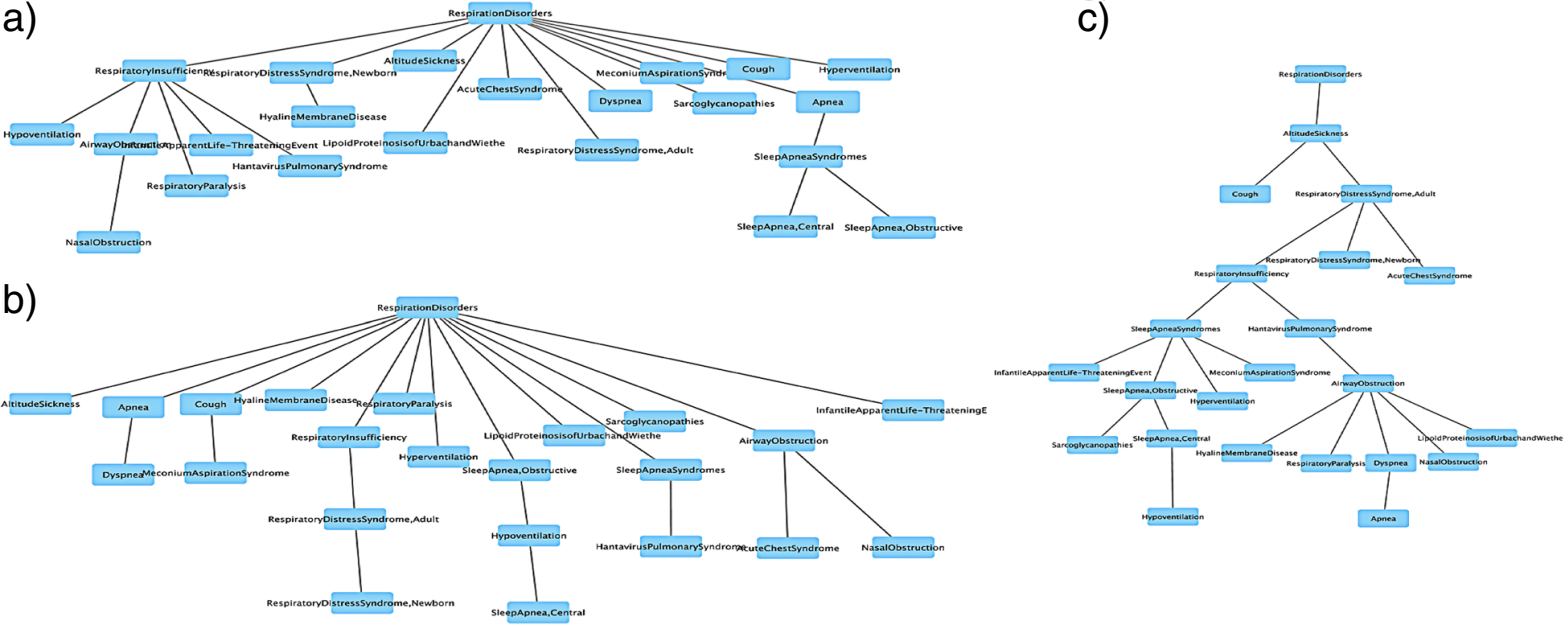
A MeSH tree rooted at “Respiration Disorder” and corresponding inferred disease trees. **a** The MeSH tree containing “Respiration Disorder” and its descendants. **b** The disease tree inferred by Parent Promotion on data from the tree in **a**). **c** The disease tree inferred by MWST from the same data. MWST builds a taller and slimmer tree. As a result, most diseases have more ancestors in **c**) than in **a**) or **b**). This leads MWST to have good performance with respect to Ancestor Recall (AR)

We also notice that despite its relatively poor performance overall, MWST seems to have good Ancestor Recall in many cases, sometimes even beating other methods. This may be because MWST tends to infer tall, thin trees rather than short and broad ones. Figure 4 illustrates this tendency. A node has more ancestors in tall, thin trees than in broad trees, and as a result, is more likely to share ancestors with the reference.

By attempting to infer relationships for each MeSH disease category separately, or within specific subnetworks of the Disease Ontology, most of the work described here has only a limited ability to detect novel molecular connections across diseases currently thought to be unrelated. However, we can begin to address the question of whether such discovery is possible with these methods by looking at the performance of Parent Promotion on data from the full Disease Ontology, and by examining inferred edges connecting pairs of disease terms that are not directly connected in the DO.

We found 1900 such pairs. Most of these make unsurprising connections. For example, progressive muscular atrophy was, in our inferred hierarchy, directly connected to spinal muscular atrophy because they share 34 genes (all of those associated with the first disease term). Other pairs may span different medical domains and tissues yet have well-known commonalities that are already described in existing hierarchies (e.g. rheumatoid arthritis and type I diabetes mellitus, both of which are listed as autoimmune disorders in MeSH).

However, there are other inferred edges whose relationships are plausible but not currently characterized. For example, liver cirrhosis and pre-eclampsia share an edge in our inferred hierarchy because they have large and highly overlapping sets of associated genes. These disorders initially appear to affect very different anotomical systems and processes; both the Disease Ontology and MeSH categorize pre-eclampsia under cardiovascular disease/hypertension (MeSH also lists it as a pregnancy complication), while cirrhosis is represented primarily as a liver disease in both hierarchies. Yet there is evidence that cirrhosis elevates the risk of pre-eclampsia during pregnancy [26]. There are also specific cases (e.g. HELLP syndrome, characterized by hemolysis, elevated liver enzymes, and low platelet count) that link liver dysfunction with increased pre-eclampsia risk [27]. As another example, fatty liver disease is also surprisingly linked to pterygium or “surfer’s eye,” characterized by fleshy growths of the eye that are linked to sunlight exposure. Molecular markers associated with pterygium appear to be associated with cell migration or involved with epithelial-to-mesenchymal transition (EMT) [28], a class of genes also thought to play a role in how the liver responds to injury such as that caused by fatty liver disease [29]. Future work exploring the implication of such potential connections may be warranted.

## Conclusions

We have demonstrated that it is possible to recover much of the structure of both MeSH disease trees and the DO from molecular data alone. However, this work is a preliminary analysis, and there is much more to learn.

Although our aim in this project has been only to infer gene-based relationships between disease terms in existing taxonomic systems, one ultimate goal for a 21st-century disease taxonomy is the inference of *new* disease terms based on molecular information [4, 7]. Classification of cancer or autism subtypes based on underlying genetic contributions, for example, might be possible in such a system.

The examples in the previous section of discovering links across apparently disparate disease types raise the possibility that novel connections in the inferred hierarchies for the full Disease Ontology data may correspond to novel disease subtypes with common molecular causes. Thus the discovery of new disease terms could arise from future work based on such analyses. Of the methods described here, CliXO is the only one that might *directly* address this problem, by inferring internal nodes corresponding to sets of genes and then by finding new methods to map these gene sets into plausible disease classes. Further exploration of its abilities to do so, or extension of clustering-based methods analogous to Parent Promotion to incorporate comparable possibilities, is warranted.

Taxonomy inference using data from diseases across organ systems and tissues, such as that in the full Disease Ontology data set, may also lead to improved categorization of disease processes. Subgraphs of the inferred hierarchies may represent disease groups specific to certain anatomical systems, and investigation of disease genes associated with such a subgraph might provide some insights into anatomical expression and relevance of disease genes. However, to identify inferred subgraphs representing specific anatomical systems we would need a comprehensive mapping between DO terms and these systems. The development of such a mapping and further interpretation of the substructure in such broad inferred hierarchies remains an interesting open question.

Future work may also include exploring the incorporation of tissue specific gene expression to integrate relevant tissues and organs with the molecular level data, and to look more broadly at ways to combine clinical and molecular data. We also have not yet fully explored the range of relevant tree- and DAG-inference methods from the machine-learning community. However, the current results leave us optimistic that by including molecular information, it will be possible to construct integrated disease taxonomies that better support medical research in the genomic era.

## Additional files

Additional file 1 Performance of three disease hierarchy inference algorithms (Parent Promotion, CliXO, MWST): Edge Correctness, Ancestor Correctness, Ancestor Precision/Recall and F-score for 23 MeSH trees. (PDF 78 kb)

Additional file 2 Performance of Parent Promotion using disease-gene association information in OMIM, Genopedia and combination of two: Ancestor Correctness, Ancestor Precision/Recall and F-score for 23 MeSH trees. (PDF 61 kb)

Additional file 3 Change in performance of Parent Promotion depending on the size of disease-gene association information: Edge Correctness, Ancestor Correctness, Ancestor Precision/Recall and F-score for 23 MeSH trees. (PDF 53 kb)

## Abbreviations

AC: Ancestor Correctness
AP: Ancestor precision
AR: Ancestor recall
CliXO: Clique Extracted Ontology
DAG: Directed acyclic graph
DO: Disease Ontology
EC: Edge Correctness
HP: Hierarchical precision
HR: Hierarchical recall
HuGE database: Human genome epidemiology database
ICD: International classification of diseases
MeSH: Medical subject heading
MWST: Minimum weight spanning tree
NCBI: National Center for Biotechnology Information
OMIM: Online Mendelian inheritance in man
PheWAS: Phenome wide association studies
SNOMED CT: Systematized nomenclature of medicine, clinical terms
UMLS: Unified medical language system

## References

[1] Park J, Wick HC, Kee DE, Noto K, Maron JL, Slonim DK (2014). Finding novel molecular connections between developmental processes and disease. PLoS Comput Biol.

[2] Denny JC, Ritchie MD, Basford MA, Pulley JM, Bastarache L, Brown-Gentry K, Wang D, Masys DR, Roden DM, Crawford DC (2010). PheWAS: demonstrating the feasibility of a phenome-wide scan to discover gene-disease associations. Bioinformatics.

[3] Kohler S, Bauer S, Horn D, Robinson PN (2008). Walking the interactome for prioritization of candidate disease genes. Am J Hum Genet.

[4] Desmond-Hellmann S, Sawyers CL, et al. Toward precision medicine: Building a knowledge network for biomedical research and a new taxonomy of disease. Technical report, National Research Council. 2011. 22536618

[5] Kramer M, Dutkowski J, Yu M, Bafna V, Ideker T (2014). Inferring gene ontologies from pairwise similarity data. Bioinformatics.

[6] Dutkowski J, Kramer M, Surma MA, Balakrishnan R, Cherry JM, Krogan NJ, Ideker T (2013). A gene ontology inferred from molecular networks. Nat Biotechnol.

[7] Kola I, Bell J (2011). A call to reform the taxonomy of human disease. Nat Rev Drug Discov.

[8] Smith B, Ashburner M, Rosse C, Bard J, Bug W, Ceusters W, Goldberg LJ, Eilbeck K, Ireland A, Mungall CJ, Leontis N, Rocca-Serra P, Ruttenberg A, Sansone SA, Scheuermann RH, Shah N, Whetzel PL, Lewis S (2007). The OBO Foundry: coordinated evolution of ontologies to support biomedical data integration. Nat Biotechnol.

[9] Lambe P (2007). Organising Knowledge: Taxonomies, Knowledge and Organisational Effectiveness.

[10] Lowe HJ, Barnett GO (1994). Understanding and using the medical subject headings (mesh) vocabulary to perform literature searches. JAMA.

[11] Wang AY, Sable JH, Spackman KA. The snomed clinical terms development process: Refinement and analysis of content. In: Proc AMIA Symp. American Medical Informatics Association: 2002. p. 845–9. PMC2244575 12463944

[12] Bodenreider O (2004). The Unified Medical Language System (UMLS): integrating biomedical terminology. Nucleic Acids Res.

[13] Schriml LM, Arze C, Nadendla S, Chang Y-WW, Mazaitis M, Felix V, Feng G, Kibbe WA (2011). Disease Ontology: a backbone for disease semantic integration. Nucleic Acids Res.

[14] Singh R, Xu J, Berger B (2008). Global alignment of multiple protein interaction networks with application to functional orthology detection. PNAS.

[15] Sanchez D, Batet M, Isern D, Valls A (2012). Ontology-based semantic similarity: A new feature-based approach. Expert Syst Appl.

[16] Petrakis EGM, Varelas G, Hliaoutakis A, Raftopoulou P (2006). X-similarity: Computing semantic similarity between concepts from different ontologies. J Digit Inf Manag.

[17] Verspoor K, Cohn J, Susan Mniszewski CJ (2006). A categorization approach to automated ontological function annotation. Protein Sci.

[18] Schriml LM, Arze C, Nadendla S, Chang Y-WW, Mazaitis M, Felix V, Feng G, Kibbe WA (2011). Disease Ontology: a backbone for disease semantic integration. Nucleic Acids Res.

[19] Online Mendelian Inheritance in Man, OMIM^Ⓡ^. Baltimore: McKusick-Nathans Institute of Genetic Medicine, Johns Hopkins University. https://omim.org/. Accessed 3 Feb 2016.

[20] Lin BK, Clyne M, Walsh M, Gomez O, Yu W, Gwinn M, Khoury MJ (2006). Tracking the epidemiology of human genes in the literature: the HuGE published literature database. Am J Epidemiol.

[21] Amberger J, Bocchini C, Schiettecatte F, Scott A, Hamosh A (2015). Omim.org: Online mendelian inheritance in man (omimⓇ), an online catalog of human genes and genetic disorders. Nucleic Acids Res.

[22] Davis AP, Grondin CJ, Lennon-Hopkins K, Saraceni-Richards C, Sciaky D, King BL, Wiegers TC, Mattingly CJ (2014). The Comparative Toxicogenomics Database’s 10th year anniversary: update 2015. Nucleic Acids Res.

[23] Pallejà A, Tsafou K, Binder JX, Jensen LJ, Pletscher-Frankild, Sune (2015). Diseases: text mining and data integration of disease-gene associations. Methods.

[24] Jaccard P (1901). Distribution de la flore alpine dans le bassin des drouces et dans quelques regions voisines. Bulletin de la Société Vaudoise des Sciences Naturelles.

[25] Cormen TH, Leiserson CE, Rivest RL, Stein C (2009). Introduction to Algorithms, Third Edition.

[26] Rasheed SM, Abdel Monem AM, Abd Ellah AH (2013). Prognosis and determinants of pregnancy outcome among patients with post-hepatitis liver cirrhosis. Int J Gynaecol Obstet.

[27] Munoz-Hernandez R, Medrano-Campillo P, Miranda ML, Macher HC, Praena-Fernandez JM, Vallejo-Vaz AJ, Dominguez-Simeon MJ, Moreno-Luna R, Stiefel P (2017). Total and Fetal Circulating Cell-Free DNA, Angiogenic, and Antiangiogenic Factors in Preeclampsia and HELLP Syndrome. Am J Hypertens.

[28] Jaworski CJ, Aryankalayil-John M, Campos MM, Fariss RN, Rowsey J, Agarwalla N, Reid TW, Dushku N, Cox CA, Carper D, Wistow G (2009). Expression analysis of human pterygium shows a predominance of conjunctival and limbal markers and genes associated with cell migration. Mol Vis.

[29] Choi SS, Diehl AM (2009). Epithelial-to-mesenchymal transitions in the liver. Hepatology.

[30] Shannon P, Markiel A, Ozier O, Baliga NS, Wang JT, Ramage D, Amin N, Schwikowski B, Ideker T (2003). Cytoscape: A software environment for integrated models of biomolecular interaction networks. Genome Res.

