# Supplementary material for: Towards a more molecular taxonomy of disease
Source: J Biomed Semantics. 2017 Jul 27;8:25. doi: 10.1186/s13326-017-0134-0 (PMC5530939; doi:10.1186/s13326-017-0134-0)
Supplement: Supplementary file 1 — Performance of three disease hierarchy inference algorithms (Parent Promotion, CliXO, MWST): Edge Correctness, Ancestor Correctness, Ancestor Precision/Recall and F-score for 23 MeSH trees. (PDF 78 kb) [file 13326_2017_134_MOESM1_ESM.pdf]

Full experimental results for disease hierarchy inference using the Medical Subject Headings (MeSH) trees

Table 1: **Edge Correctness (EC) and Ancestor Correctness (AC) for the MeSH subtrees used for method development.** Best performance across different inference methods is highlighted.

	Edge Correctness			Ancestor Correctness		
Root Disease	Parent Promotion	CliXO	MWST	Parent Promotion	CliXO	MWST
Infant, Premature, Diseases	0.19	**0.40**	0.20	**0.60**	**0.60**	0.57
Dementia	0.50	**0.75**	0.25	0.67	**0.84**	0.39
Respiration Disorder	0.36	**0.50**	0.14	0.52	**0.63**	0.34
Eye Diseases	**0.19**	0.13	0.06	**0.45**	0.32	0.12

Table 2: **F-score with Ancestor Precision (AP) and Ancestor Recall (AR) for the MeSH subtrees used for method development.** Best performance across different inference methods is highlighted.

	(AP, AR) F-score					
Root Disease	Parent Promotion		CliXO		MWST	
Infant, Premature, Diseases	(**0.70** , **0.90**)	**0.79**	(0.60 , 0.80)	0.69	(0.67 , **0.90**)	0.77
Dementia	(0.70 , 0.86)	0.77	(**0.85** , **0.94**)	**0.90**	(0.41 , 0.82)	0.55
Repiration Disorders	(0.62 , 0.79)	0.69	(**0.69** , 0.85)	**0.76**	(0.35 , **0.92**)	0.50
Eye Diseases	(**0.64** , **0.64**)	**0.64**	(0.41 , 0.53)	0.46	(0.14 , 0.60)	0.23

Table 3: **23 MeSH disease trees of various sizes and size of corresponding disease hierarchies inferred by CliXO.** We tested our inference methods by building disease hierarchies for the MeSH terms belonging to 23 different trees. While organizing disease terms in forms of trees, the MeSH allows multiple nodes to have same disease name. We treat these nodes as they are the same node and therefore, the MeSH trees are rather DAGs than trees. As shown below, most MeSH trees with n nodes have more than $n-1$ edges after collapsing duplicate nodes. The table also reports the number of nodes and edges created by CliXO. Unlike Parent Promotion and MWST, build trees with the same set of disease nodes as the reference, CliXO builds a DAG and does not neccessarily have the same set of disease nodes as reference.

	MeSH trees		Inferred hierarchies by CliXO	
Root Disease	#Diseases (Nodes)	#Edges	#Diseases (Nodes)	#Edges
Bacterial Infections and Mycoses	142	141	114	158
Virus Diseases	86	85	165	365
Parasitic Diseases	37	36	66	93
Neoplasms	402	401	457	806
Musculoskeletal Diseases	227	226	343	696
Digestive System Diseases	179	178	314	650
Stomatognathic Diseases	111	110	167	290
Respiratory Tract Diseases	126	125	261	539
Otorhinolaryngologic Diseases	65	64	132	181
Nervous System Diseases	566	565	611	1124
Male Urogenital Diseases	138	137	282	654
Female Urogenital Diseases and Pregnancy Complications	203	202	301	501
Cardiovascular Diseases	266	265	364	776
Hemic and Lymphatic Diseases	197	196	281	464
Congenital, Hereditary, and Neonatal Diseases and Abnormalities	579	578	664	1167
Skin and Connective Tissue Diseases	240	239	258	439
Nutritional and Metabolic Diseases	282	281	440	817
Endocrine System Diseases	121	120	323	833
Immune System Diseases	163	162	293	560
Pathological Conditions, Signs and Symptoms	447	446	584	1581
Occupational Diseases	15	14	36	49
Chemically-Induced Disorders	53	52	150	277
Wounds and Injuries	74	73	107	165

Table 4: **Edge correctness (EC) for 23 MeSH disease trees.** Best performance across different inference methods is highlighted.

	Edge Correctness		
Root Disease	Parent Promotion	CliXO	MWST
Bacterial Infections and Mycoses	**0.05**	**0.05**	0.01
Virus Diseases	**0.19**	0.14	0.09
Parasitic Diseases	**0.26**	0.23	0.09
Neoplasms	**0.08**	0.04	0.03
Musculoskeletal Diseases	**0.12**	0.06	0.05
Digestive System Diseases	**0.14**	0.11	0.03
Stomatognathic Diseases	0.05	**0.17**	0.04
Respiratory Tract Diseases	**0.15**	0.14	0.04
Otorhinolaryngologic Diseases	0.12	**0.17**	0.08
Nervous System Diseases	**0.09**	0.06	0.04
Male Urogenital Diseases	**0.09**	0.08	**0.09**
Female Urogenital Diseases and Pregnancy Complications	**0.13**	0.08	0.07
Cardiovascular Diseases	**0.07**	0.04	0.04
Hemic and Lymphatic Diseases	0.08	**0.10**	0.04
Congenital, Hereditary, and Neonatal Diseases and Abnormalities	**0.09**	0.06	0.06
Skin and Connective Tissue Diseases	**0.13**	0.06	0.07
Nutritional and Metabolic Diseases	**0.10**	0.06	0.05
Endocrine System Diseases	**0.22**	0.15	0.14
Immune System Diseases	**0.11**	0.08	0.04
Pathological Conditions, Signs and Symptoms	**0.10**	0.02	0.04
Occupational Diseases	0.29	**0.50**	0.21
Chemically-Induced Disorders	**0.18**	0.15	0.15
Wounds and Injuries	**0.23**	0.20	0.05
Average	**0.13**	0.12	0.07
Standard Deviation	0.06	0.10	0.04

Table 5: **Ancestor correctness (AC) for 23 MeSH disease trees.** Best performance across different inference methods is highlighted.

	Ancestor Correctness		
Root Disease	Parent Promotion	CliXO	MWST
Bacterial Infections and Mycoses	**0.15**	0.11	0.08
Virus Diseases	**0.36**	0.29	0.16
Parasitic Diseases	**0.47**	0.46	0.23
Neoplasms	**0.29**	0.10	0.08
Musculoskeletal Diseases	**0.36**	0.21	0.11
Digestive System Diseases	**0.39**	0.26	0.10
Stomatognathic Diseases	**0.22**	0.18	0.09
Respiratory Tract Diseases	**0.37**	0.30	0.11
Otorhinolaryngologic Diseases	**0.24**	0.21	0.10
Nervous System Diseases	**0.29**	0.16	0.06
Male Urogenital Diseases	**0.32**	0.24	0.11
Female Urogenital Diseases and Pregnancy Complications	**0.19**	0.15	0.06
Cardiovascular Diseases	**0.28**	0.14	0.08
Hemic and Lymphatic Diseases	**0.24**	0.17	0.07
Congenital, Hereditary, and Neonatal Diseases and Abnormalities	**0.27**	0.15	0.05
Skin and Connective Tissue Diseases	**0.34**	0.14	0.09
Nutritional and Metabolic Diseases	**0.34**	0.18	0.11
Endocrine System Diseases	**0.39**	0.26	0.10
Immune System Diseases	**0.35**	0.17	0.13
Pathological Conditions, Signs and Symptoms	**0.05**	0.00	0.01
Occupational Diseases	0.21	**0.60**	0.40
Chemically-Induced Disorders	**0.29**	0.20	0.14
Wounds and Injuries	**0.50**	0.32	0.14
Average	**0.30**	0.22	0.11
Standard Deviation	0.10	0.12	0.07

Table 6: **Ancestor Precision (AP), Ancestor Recall (AR) and F-score for 23 MeSH disease trees.** Best performance across different inference methods is highlighted.

Root Disease	(AP, AR) F-score		
	Parent Promotion	CliXO	MWST
Bacterial Infections and Mycoses	(**0.21** , 0.28) **0.24**	(0.13 , 0.24) 0.17	(0.09 , **0.29**) 0.14
Virus Diseases	(**0.47** , 0.67) **0.55**	(0.36 , 0.57) 0.44	(0.17 , **0.69**) 0.28
Parasitic Diseases	(**0.58** , **0.76**) **0.66**	(0.55 , 0.67) 0.61	(0.26 , 0.74) 0.39
Neoplasms	(**0.46** , 0.48) **0.47**	(0.13 , 0.23) 0.16	(0.10 , **0.51**) 0.16
Musculoskeletal Diseases	(**0.67** , **0.47**) **0.55**	(0.36 , 0.39) 0.38	(0.14 , 0.44) 0.21
Digestive System Diseases	(**0.58** , **0.56**) **0.57**	(0.37 , 0.39) 0.38	(0.13 , 0.40) 0.19
Stomatognathic Diseases	(**0.27** , **0.49**) **0.35**	(0.21 , 0.34) 0.26	(0.10 , 0.36) 0.15
Respiratory Tract Diseases	(**0.55** , **0.60**) **0.57**	(0.39 , 0.52) 0.45	(0.16 , 0.55) 0.24
Otorhinolaryngologic Diseases	(**0.34** , 0.40) **0.37**	(0.28 , 0.37) 0.31	(0.13 , **0.43**) 0.20
Nervous System Diseases	(**0.56** , **0.43**) **0.49**	(0.25 , 0.31) 0.27	(0.07 , 0.42) 0.12
Male Urogenital Diseases	(**0.62** , **0.44**) **0.52**	(0.43 , 0.42) 0.43	(0.14 , 0.39) 0.21
Female Urogenital Diseases and Pregnancy Complications	(**0.30** , **0.29**) **0.29**	(0.21 , 0.28) 0.24	(0.08 , 0.28) 0.12
Cardiovascular Diseases	(**0.51** , **0.42**) **0.46**	(0.24 , 0.24) 0.24	(0.10 , 0.39) 0.16
Hemic and Lymphatic Diseases	(**0.36** , **0.46**) **0.40**	(0.25 , 0.31) 0.28	(0.08 , 0.46) 0.13
Congenital, Hereditary, and Neonatal Diseases and Abnormalities	(**0.48** , **0.37**) **0.42**	(0.25 , 0.29) 0.27	(0.06 , 0.35) 0.10
Skin and Connective Tissue Diseases	(**0.51** , 0.50) **0.50**	(0.20 , 0.30) 0.24	(0.10 , **0.55**) 0.17
Nutritional and Metabolic Diseases	(**0.65** , **0.43**) **0.52**	(0.31 , 0.29) 0.30	(0.14 , 0.42) 0.21
Endocrine System Diseases	(**0.54** , **0.62**) **0.57**	(0.34 , 0.47) 0.40	(0.12 , 0.57) 0.20
Immune System Diseases	(**0.60** , **0.47**) **0.53**	(0.27 , 0.30) 0.28	(0.16 , 0.46) 0.24
Pathological Conditions, Signs and Symptoms	(**0.06** , **0.10**) **0.07**	(0.00 , 0.02) 0.01	(0.01 , 0.07) 0.02
Occupational Diseases	(0.24 , 0.37) 0.29	(**0.67** , 0.89) **0.77**	(0.44 , **0.93**) 0.60
Chemically-Induced Disorders	(**0.41** , 0.52) **0.46**	(0.25 , 0.40) 0.31	(0.17 , **0.54**) 0.26
Wounds and Injuries	(**0.59** , **0.74**) **0.66**	(0.38 , 0.55) 0.45	(0.16 , 0.73) 0.26
Average	(**0.46** , 0.47) **0.47**	(0.30 , 0.38) 0.33	(0.13 , **0.48**) 0.21
Standard Deviation	(0.16 , 0.14) 0.15	(0.14 , 0.17) 0.15	(0.08 , 0.18) 0.11
