# Supplementary material for: Towards a more molecular taxonomy of disease
Source: J Biomed Semantics. 2017 Jul 27;8:25. doi: 10.1186/s13326-017-0134-0 (PMC5530939; doi:10.1186/s13326-017-0134-0)
Supplement: Supplementary file 2 — Performance of Parent Promotion using disease-gene association information in OMIM, Genopedia and combination of two: Ancestor Correctness, Ancestor Precision/Recall and F-score for 23 MeSH trees. (PDF 61 kb) [file 13326_2017_134_MOESM2_ESM.pdf]

Performance of Parent Promotion using disease-gene association information in two different data sources: Online Mendelian Inheritance in Man (OMIM) and Genopedia

Table 1: **The amount of disease-gene association data included in OMIM and Genopedia** The number of association pairs covered by OMIM and Genopedia is reported along with the number of genes and diseases in the pairs. The amount of disease-gene association information in the combination of OMIM and Genopedia is also included for comparison.

	OMIM			Genopedia			Combined		
Root Disease	disease	gene	pair	disease	gene	pair	disease	gene	pair
Bacterial Infections and Mycoses	15	29	47	138	876	2,891	142	885	2,918
Virus Diseases	11	29	31	86	2,583	5,287	86	2,589	5,299
Parasitic Diseases	1	17	17	37	296	704	37	298	709
Neoplasms	128	253	455	389	5,110	31,190	402	5,157	31,385
Musculoskeletal Diseases	154	499	763	177	2,605	5,333	227	2,839	5,921
Digestive System Diseases	61	180	212	175	3,478	11,604	179	3,528	11,698
Stomatognathic Diseases	28	74	92	99	1,389	3,641	111	1,416	3,702
Respiratory Tract Diseases	29	89	100	99	2,402	6,759	103	2,432	6,812
Otorhinolaryngologic Diseases	23	149	171	60	812	1,670	65	909	1,810
Nervous System Diseases	286	881	1,248	490	3,726	12,239	553	4,079	13,125
Male Urogenital Diseases	73	221	271	126	2,998	7,055	138	3,069	7,219
Female Urogenital Diseases and Pregnancy Complications	80	232	286	190	3,459	11,010	203	3,528	11,189
Cardiovascular Diseases	96	248	338	250	4,319	15,052	266	4,370	15,206
Hemic and Lymphatic Diseases	107	249	294	174	1,819	5,963	197	1,915	6,150
Congenital, Hereditary, and Neonatal Diseases and Abnormalities	501	1,541	2,255	412	2,899	7,429	573	3,599	9,029
Skin and Connective Tissue Diseases	136	337	431	188	3,095	7,565	240	3,234	7,856
Nutritional and Metabolic Diseases	222	695	832	218	4,816	11,919	282	5,070	12,452
Endocrine System Diseases	75	228	281	113	4,700	10,563	121	4,743	10,700
Immune System Diseases	56	197	224	151	4,294	11,806	163	4,330	11,924
Pathological Conditions, Signs and Symptoms	130	497	633	431	10,348	31,086	447	10,442	31,568
Occupational Diseases	1	1	1	15	441	559	15	441	559
Chemically-Induced Disorders	6	16	16	53	4,096	5,750	53	4,099	5,754
Wounds and Injuries	4	4	4	72	490	1,000	74	493	1,003
Total	1,173	2,434	4,878	2,499	12,527	137,718	2,755	12,873	140,928

Table 2: **Size of reference and inferred disease hierarchies using OMIM and Genopedia.** The size of disease hierarchies inferred using disease-gene association information in OMIM and Genopedia is reported in terms of number of nodes and edges. The size of reference and inference made using both OMIM and Genopedia is reported together for comparison.

	OMIM		Genopedia		Combined		Reference	
Root Disease	nodes	edges	nodes	edges	nodes	edges	nodes	edges
Bacterial Infections and Mycoses	15	14	138	137	142	141	142	179
Virus Diseases	11	10	86	85	86	85	86	112
Parasitic Diseases	0	0	37	36	37	36	37	43
Neoplasms	128	127	389	388	402	401	402	511
Musculoskeletal Diseases	154	153	177	176	227	226	227	279
Digestive System Diseases	61	60	175	174	179	178	179	232
Stomatognathic Diseases	28	27	99	98	111	110	111	120
Respiratory Tract Diseases	29	28	99	98	103	102	103	131
Otorhinolaryngologic Diseases	23	22	60	59	65	64	65	60
Nervous System Diseases	286	285	490	489	553	552	553	723
Male Urogenital Diseases	73	72	126	125	138	137	138	159
Female Urogenital Diseases and Pregnancy Complications	80	79	190	189	203	202	203	231
Cardiovascular Diseases	96	95	250	249	266	265	266	323
Hemic and Lymphatic Diseases	107	106	174	173	197	196	197	265
Congenital, Hereditary, and Neonatal Diseases and Abnormalities	501	500	412	411	573	572	573	807
Skin and Connective Tissue Diseases	136	135	188	187	240	239	240	301
Nutritional and Metabolic Diseases	222	221	218	217	282	281	282	388
Endocrine System Diseases	75	74	113	112	121	120	121	140
Immune System Diseases	56	55	151	150	163	162	163	171
Pathological Conditions, Signs and Symptoms	130	129	431	430	447	446	447	303
Occupational Diseases	0	0	15	14	15	14	15	14
Chemically-Induced Disorders	6	5	53	52	53	52	53	55
Wounds and Injuries	4	3	72	71	74	73	74	88

Table 3: **Ancestor correctness (AC) for 23 MeSH disease trees.**

Root Disease	Ancestor Correctness		
	OMIM	Genopedia	Combined
Bacterial Infections and Mycoses	0.03	0.17	0.15
Virus Diseases	0.00	0.37	0.36
Parasitic Diseases	0.00	0.47	0.47
Neoplasms	0.01	0.29	0.29
Musculoskeletal Diseases	0.26	0.29	0.36
Digestive System Diseases	0.05	0.38	0.39
Stomatognathic Diseases	0.02	0.20	0.22
Respiratory Tract Diseases	0.15	0.40	0.37
Otorhinolaryngologic Diseases	0.09	0.23	0.24
Nervous System Diseases	0.17	0.27	0.29
Male Urogenital Diseases	0.17	0.32	0.32
Female Urogenital Diseases and Pregnancy Complications	0.11	0.20	0.19
Cardiovascular Diseases	0.07	0.33	0.28
Hemic and Lymphatic Diseases	0.19	0.21	0.24
Congenital, Hereditary, and Neonatal Diseases and Abnormalities	0.24	0.20	0.27
Skin and Connective Tissue Diseases	0.18	0.27	0.34
Nutritional and Metabolic Diseases	0.01	0.28	0.34
Endocrine System Diseases	0.29	0.36	0.39
Immune System Diseases	0.14	0.31	0.35
Pathological Conditions, Signs and Symptoms	0.01	0.05	0.05
Occupational Diseases	0.00	0.21	0.21
Chemically-Induced Disorders	0.01	0.29	0.29
Wounds and Injuries	0.00	0.46	0.50
Average	0.10	0.29	0.30
Standard Deviation	0.09	0.09	0.10

Table 4: **Ancestor Precision (AP), Ancestor Recall (AR) and F-score for 23 MeSH disease trees.**

	(AP, AR) F-score		
Root Disease	OMIM	Genopedia	Combined
Bacterial Infections and Mycoses	(0.03 , 0.04) 0.04	(0.23 , 0.30) 0.26	(0.21 , 0.28) 0.24
Virus Diseases	(0.00 , 0.00) 0.00	(0.50 , 0.66) 0.57	(0.47 , 0.67) 0.55
Parasitic Diseases	(0.00 , 0.00) 0.00	(0.58 , 0.76) 0.66	(0.58 , 0.76) 0.66
Neoplasms	(0.01 , 0.01) 0.01	(0.46 , 0.47) 0.46	(0.46 , 0.48) 0.47
Musculoskeletal Diseases	(0.50 , 0.32) 0.39	(0.51 , 0.42) 0.46	(0.67 , 0.47) 0.55
Digestive System Diseases	(0.10 , 0.07) 0.08	(0.57 , 0.55) 0.56	(0.58 , 0.56) 0.57
Stomatognathic Diseases	(0.03 , 0.06) 0.04	(0.25 , 0.43) 0.32	(0.27 , 0.49) 0.35
Respiratory Tract Diseases	(0.17 , 0.24) 0.20	(0.59 , 0.56) 0.58	(0.55 , 0.60) 0.57
Otorhinolaryngologic Diseases	(0.17 , 0.11) 0.13	(0.32 , 0.38) 0.35	(0.34 , 0.40) 0.37
Nervous System Diseases	(0.35 , 0.23) 0.28	(0.50 , 0.40) 0.44	(0.56 , 0.43) 0.49
Male Urogenital Diseases	(0.32 , 0.23) 0.27	(0.62 , 0.38) 0.47	(0.62 , 0.44) 0.52
Female Urogenital Diseases and Pregnancy Complications	(0.14 , 0.17) 0.15	(0.28 , 0.30) 0.29	(0.30 , 0.29) 0.29
Cardiovascular Diseases	(0.12 , 0.13) 0.13	(0.51 , 0.47) 0.49	(0.51 , 0.42) 0.46
Hemic and Lymphatic Diseases	(0.33 , 0.23) 0.27	(0.31 , 0.37) 0.34	(0.36 , 0.46) 0.40
Congenital, Hereditary, and Neonatal Diseases and Abnormalities	(0.44 , 0.31) 0.36	(0.30 , 0.35) 0.33	(0.48 , 0.37) 0.42
Skin and Connective Tissue Diseases	(0.28 , 0.28) 0.28	(0.40 , 0.41) 0.40	(0.51 , 0.50) 0.50
Nutritional and Metabolic Diseases	(0.03 , 0.02) 0.02	(0.48 , 0.35) 0.40	(0.65 , 0.43) 0.52
Endocrine System Diseases	(0.42 , 0.37) 0.39	(0.50 , 0.56) 0.53	(0.54 , 0.62) 0.57
Immune System Diseases	(0.24 , 0.18) 0.21	(0.53 , 0.43) 0.48	(0.60 , 0.47) 0.53
Pathological Conditions, Signs and Symptoms	(0.01 , 0.02) 0.01	(0.06 , 0.10) 0.07	(0.06 , 0.10) 0.07
Occupational Diseases	(0.00 , 0.00) 0.00	(0.24 , 0.37) 0.29	(0.24 , 0.37) 0.29
Chemically-Induced Disorders	(0.02 , 0.01) 0.01	(0.41 , 0.52) 0.46	(0.41 , 0.52) 0.46
Wounds and Injuries	(0.00 , 0.00) 0.00	(0.56 , 0.71) 0.63	(0.59 , 0.74) 0.66
Average	(0.16 , 0.13) 0.14	(0.42 , 0.44) 0.43	(0.46 , 0.47) 0.47
Standard Deviation	(0.16 , 0.12) 0.14	(0.14 , 0.14) 0.14	(0.16 , 0.14) 0.15
