# Supplementary material for: Towards a more molecular taxonomy of disease
Source: J Biomed Semantics. 2017 Jul 27;8:25. doi: 10.1186/s13326-017-0134-0 (PMC5530939; doi:10.1186/s13326-017-0134-0)
Supplement: Supplementary file 3 — Change in performance of Parent Promotion depending on the size of disease-gene association information: Edge Correctness, Ancestor Correctness, Ancestor Precision/Recall and F-score for 23 MeSH trees. (PDF 53 kb) [file 13326_2017_134_MOESM3_ESM.pdf]

Change in performance of Parent Promotion depending on the size of available disease-gene association information

Table 1: **Edge correctness (EC) for 23 MeSH disease trees.**

	Edge Correctness		
Root Disease	50% withheld	25% withheld	None withheld
Bacterial Infections and Mycoses	0.06	0.06	0.05
Virus Diseases	0.15	0.16	0.19
Parasitic Diseases	0.24	0.32	0.26
Neoplasms	0.07	0.08	0.08
Musculoskeletal Diseases	0.08	0.10	0.12
Digestive System Diseases	0.09	0.11	0.14
Stomatognathic Diseases	0.08	0.08	0.05
Respiratory Tract Diseases	0.15	0.13	0.16
Otorhinolaryngologic Diseases	0.12	0.15	0.12
Nervous System Diseases	0.06	0.09	0.09
Male Urogenital Diseases	0.10	0.11	0.09
Female Urogenital Diseases and Pregnancy Complications	0.08	0.11	0.13
Cardiovascular Diseases	0.07	0.08	0.07
Hemic and Lymphatic Diseases	0.07	0.08	0.08
Congenital, Hereditary, and Neonatal Diseases and Abnormalities	0.06	0.08	0.10
Skin and Connective Tissue Diseases	0.09	0.12	0.13
Nutritional and Metabolic Diseases	0.08	0.08	0.10
Endocrine System Diseases	0.18	0.19	0.22
Immune System Diseases	0.10	0.10	0.11
Pathological Conditions, Signs and Symptoms	0.06	0.08	0.10
Occupational Diseases	0.21	0.18	0.29
Chemically-Induced Disorders	0.15	0.15	0.18
Wounds and Injuries	0.12	0.20	0.23
Average	0.11	0.12	0.13
Standard Deviation	0.05	0.06	0.06

Table 2: **Ancestor correctness (AC) for 23 MeSH disease trees.** Best performance across different inference methods is highlighted.

Root Disease	Ancestor Correctness		
	50% withheld	25% withheld	None withheld
Bacterial Infections and Mycoses	0.17	0.19	0.15
Virus Diseases	0.35	0.38	0.36
Parasitic Diseases	0.49	0.51	0.47
Neoplasms	0.28	0.30	0.29
Musculoskeletal Diseases	0.31	0.33	0.36
Digestive System Diseases	0.29	0.34	0.39
Stomatognathic Diseases	0.20	0.22	0.22
Respiratory Tract Diseases	0.41	0.40	0.37
Otorhinolaryngologic Diseases	0.22	0.26	0.24
Nervous System Diseases	0.26	0.29	0.29
Male Urogenital Diseases	0.30	0.36	0.32
Female Urogenital Diseases and Pregnancy Complications	0.19	0.21	0.19
Cardiovascular Diseases	0.30	0.30	0.28
Hemic and Lymphatic Diseases	0.22	0.24	0.24
Congenital, Hereditary, and Neonatal Diseases and Abnormalities	0.22	0.25	0.27
Skin and Connective Tissue Diseases	0.26	0.29	0.34
Nutritional and Metabolic Diseases	0.27	0.31	0.34
Endocrine System Diseases	0.39	0.39	0.39
Immune System Diseases	0.32	0.34	0.35
Pathological Conditions, Signs and Symptoms	0.03	0.04	0.05
Occupational Diseases	0.14	0.10	0.21
Chemically-Induced Disorders	0.28	0.27	0.29
Wounds and Injuries	0.35	0.43	0.50
Average	0.27	0.29	0.30
Standard Deviation	0.09	0.10	0.10

Table 3: **Ancestor Precision (AP), Ancestor Recall (AR) and F-score for 23 MeSH disease trees.** Best performance across different inference methods is highlighted.

	(AP, AR) F-score		
Root Disease	50% withheld	25% withheld	None withheld
Bacterial Infections and Mycoses	(0.24 , 0.30) 0.27	(0.26 , 0.33) 0.29	(0.21 , 0.28) 0.24
Virus Diseases	(0.48 , 0.61) 0.00	(0.53 , 0.65) 0.58	(0.47 , 0.67) 0.55
Parasitic Diseases	(0.59 , 0.73) 0.00	(0.62 , 0.75) 0.68	(0.58 , 0.76) 0.66
Neoplasms	(0.46 , 0.42) 0.44	(0.48 , 0.46) 0.47	(0.46 , 0.48) 0.47
Musculoskeletal Diseases	(0.61 , 0.39) 0.48	(0.64 , 0.44) 0.52	(0.67 , 0.47) 0.55
Digestive System Diseases	(0.50 , 0.44) 0.47	(0.54 , 0.49) 0.51	(0.58 , 0.56) 0.57
Stomatognathic Diseases	(0.26 , 0.38) 0.31	(0.27 , 0.45) 0.34	(0.27 , 0.49) 0.35
Respiratory Tract Diseases	(0.61 , 0.54) 0.57	(0.61 , 0.56) 0.59	(0.55 , 0.60) 0.57
Otorhinolaryngologic Diseases	(0.33 , 0.29) 0.31	(0.36 , 0.37) 0.36	(0.34 , 0.40) 0.37
Nervous System Diseases	(0.50 , 0.36) 0.42	(0.56 , 0.41) 0.47	(0.56 , 0.43) 0.49
Male Urogenital Diseases	(0.56 , 0.39) 0.46	(0.60 , 0.47) 0.53	(0.62 , 0.44) 0.52
Female Urogenital Diseases and Pregnancy Complications	(0.29 , 0.28) 0.29	(0.28 , 0.33) 0.30	(0.30 , 0.29) 0.29
Cardiovascular Diseases	(0.55 , 0.39) 0.46	(0.54 , 0.42) 0.47	(0.51 , 0.42) 0.46
Hemic and Lymphatic Diseases	(0.35 , 0.35) 0.35	(0.36 , 0.38) 0.37	(0.36 , 0.46) 0.40
Congenital, Hereditary, and Neonatal Diseases and Abnormalities	(0.38 , 0.29) 0.33	(0.43 , 0.37) 0.40	(0.48 , 0.37) 0.42
Skin and Connective Tissue Diseases	(0.41 , 0.41) 0.41	(0.44 , 0.46) 0.45	(0.51 , 0.50) 0.50
Nutritional and Metabolic Diseases	(0.53 , 0.35) 0.42	(0.59 , 0.40) 0.48	(0.65 , 0.43) 0.52
Endocrine System Diseases	(0.56 , 0.55) 0.55	(0.55 , 0.58) 0.56	(0.54 , 0.62) 0.57
Immune System Diseases	(0.51 , 0.47) 0.49	(0.56 , 0.49) 0.52	(0.60 , 0.47) 0.53
Pathological Conditions, Signs and Symptoms	(0.04 , 0.06) 0.05	(0.05 , 0.08) 0.06	(0.06 , 0.10) 0.07
Occupational Diseases	(0.14 , 0.27) 0.00	(0.15 , 0.17) 0.16	(0.24 , 0.37) 0.29
Chemically-Induced Disorders	(0.37 , 0.41) 0.39	(0.37 , 0.46) 0.41	(0.41 , 0.52) 0.46
Wounds and Injuries	(0.43 , 0.62) 0.00	(0.52 , 0.71) 0.60	(0.59 , 0.74) 0.66
Average	(0.42 , 0.41) 0.41	(0.45 , 0.44) 0.44	(0.46 , 0.47) 0.46
Standard Deviation	(0.15 , 0.14) 0.14	(0.16 , 0.15) 0.14	(0.16 , 0.14) 0.14
